# A Peculiar Presentation of a Debilitating Condition: Calciphylaxis

**DOI:** 10.7759/cureus.53457

**Published:** 2024-02-02

**Authors:** Eliza Apostol, Anca Cojocaru, Ana Ion, Alexandra Maria Dorobantu, Olguta Anca Orzan

**Affiliations:** 1 Dermatology, Elias Emergency University Hospital, Bucharest, ROU

**Keywords:** vascular calcifications, necrotic skin lesions, dialysis, end-stage renal disease, calciphylaxis

## Abstract

Calciphylaxis is a debilitating condition associated with significant morbidity and mortality, often associated with patients with end-stage renal disease, in which the calcification of cutaneous arterioles and small arteries occurs, leading to subsequent ischemia and cutaneous infarction. Herein, we report the case of a diabetic patient with end-stage kidney disease on dialysis, presenting multiple intensely painful necrotic plaques on the lower extremities. The suspicion of calciphylaxis was raised based on the patient's medical history and clinical presentation, subsequently confirmed by radiological examination, which revealed calcifications along vascular pathways. Diagnosis can be established based on clinical and paraclinical grounds alone, and some clinicians may forego skin biopsy and initiate treatment presumptively. The management of calciphylaxis remains a challenge and requires a multidisciplinary approach since most patients experience intense pain that is often unresponsive to conventional analgesics, leading to a reduced quality of life.

## Introduction

Calciphylaxis represents calcific uremic arteriolopathy, a potentially devastating condition that could occur in patients with end-stage renal disease, specifically in the population undergoing dialysis with an incidence that ranges from 0.04% to 4% [1]. Occasionally, it can also occur in patients without renal impairment. The pathogenesis involves the calcification of cutaneous arterioles, leading to tissue ischemia and areas of cutaneous infarction. This condition presents high morbidity due to extremely painful and non-healing cutaneous lesions, leading to frequent hospitalizations. The mortality rate is significant, even when the condition is identified in its early stages (nearly 50% mortality rate within a year), mainly due to infectious complications in the context of compromised skin barrier leading to septicemia [1].

Risk factors associated with calciphylaxis are heterogeneous and include female gender, the presence of comorbidities such as chronic kidney disease, obesity, diabetes mellitus, liver impairment, hypoalbuminemia, and dialysis sessions. The condition is also commonly associated with autoimmune diseases such as systemic lupus erythematosus, antiphospholipid syndrome, and rheumatoid arthritis. Furthermore, the development of calciphylaxis may be facilitated by an abnormal calcium and phosphorus metabolism, which includes hyperphosphatemia, hyperparathyroidism, hypercalcemia, adynamic bone disease, or the administration of medications such as warfarin, corticosteroids, calcium binders, vitamin D analogs or hypercoagulable state like tissue trauma following the administration of subcutaneous injectable substances, such as insulin [2,3]. 

The pathophysiology of the disease involves the calcification of the medial wall of arterioles and small arteries, as well as the development of intimal fibrosis, causing reduced blood flow through endothelial dysfunction and thrombotic occlusion due to progressive calcification. These phenomena subsequently induce tissue ischemia with the appearance of necrosis and ulcers. Possible triggers are the increased calcium x phosphorus ratio and/or the elevation of parathyroid hormone (PTH) levels, commonly observed in dialysis patients due to the administration of active vitamin D. These patients may present abnormalities in bone mineral parameters. Still, these abnormal levels do not necessarily induce calciphylaxis. Conversely, sometimes calciphylaxis does not exhibit an increased calcium x phosphorus ratio or hyperparathyroidism [2-4]. 

## Case presentation

**Figure 1:**
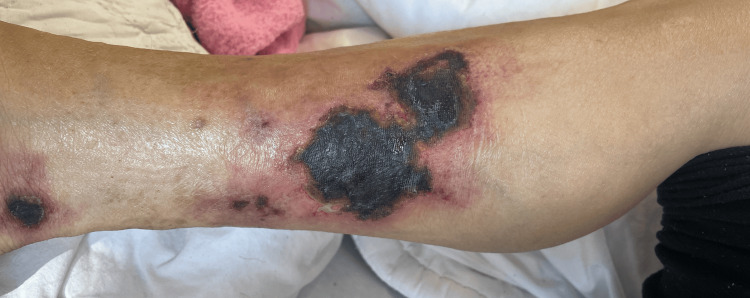
Large necrotic ulcers located on the distal end of the limb

We present the case of a 58-year-old female with multiple comorbidities who was admitted to our clinic for the appearance of intensely painful necrotic lesions on both legs three weeks before admission. She had a normal body mass index of 18.7 kg/m^2^. The patient's medical history revealed type 2 diabetes mellitus and end-stage chronic kidney disease, with the patient undergoing hemodialysis for five years. Dermatological examination revealed multiple indurated plaques overlaying livedo racemosa, progressing to necrotic, intensely painful ulcers with a star-like appearance (Figure 1).

The patient underwent several investigations, including vascular surgery consultation and a Doppler ultrasound examination. The Doppler ultrasound revealed an atheroma in the right pelvic limb; however, it did not cause any hemodynamic issues, and the Doppler signal was within optimal parameters. Additionally, the examination detected that the common femoral artery, superficial femoral artery, deep femoral artery and popliteal artery were all patent distally. Laboratory investigations showed renal anemia with liver function tests, metabolic tests, albumin level and parathyroid hormone values within normal limits (Table 1).

**Table 1:** Laboratory test results

Analysis	Result	Normal Value
Erythrocyte sedimentation rate (at one hour)	86 mm/h	0-15 mm/h
Hemoglobin (Hb)	10.8 g/dl	12.1-17.2 g/dl
Hematocrit	32.6%	36.1-50.3%
Red blood cell count	3.32x10^3^/uL	3.9-5.7x10^3^/uL
White blood cell count	11.28x10^3^/uL	3.6-9.6x10^3^/uL
Neutrophil count	8.86x10^3^/uL	1.4-6.5x10^3^/uL
Basophil count	0.02x10^3^/uL	0-0.2x10^3^/uL
Eosinophil count	0.32x10^3^/uL	0.0-0.7x10^3^/uL
Monocyte count	0.52x10^3^/uL	0-0.7x10^3^/uL
Platelet count	300x10^3^/uL	200-400x10^3^/uL
Sodium	141 mmol/L	137-145 mmol/L
Potassium	4,1 mmol/L	3.6-5 mmol/L
Calcium	2.3 mmol/L	2.2-2.7 mmol/L
Phosphorus	1.2 mmol/L	0.97-1.45 mmol/L
Creatinine	7.98 mg/dl	0.66-1.25 mg/dl
Urea	108 mg/dl	18-55 mg/dl
Albumin	3.6 g/dl	3.5-5.6 g/dl
Aspartate transaminase	14 U/L	17-59 U/L
Alanine transaminase	17 U/L	4-50 U/L
Gamma glutamyltransferase	60 U/L	15-73 U/L

Correlating the patient's medical history of end-stage chronic kidney disease with hemodialysis sessions and the clinical manifestations, including necrotic plaques associated with significant pain, raised suspicion of calciphylaxis.

**Figure 2:**
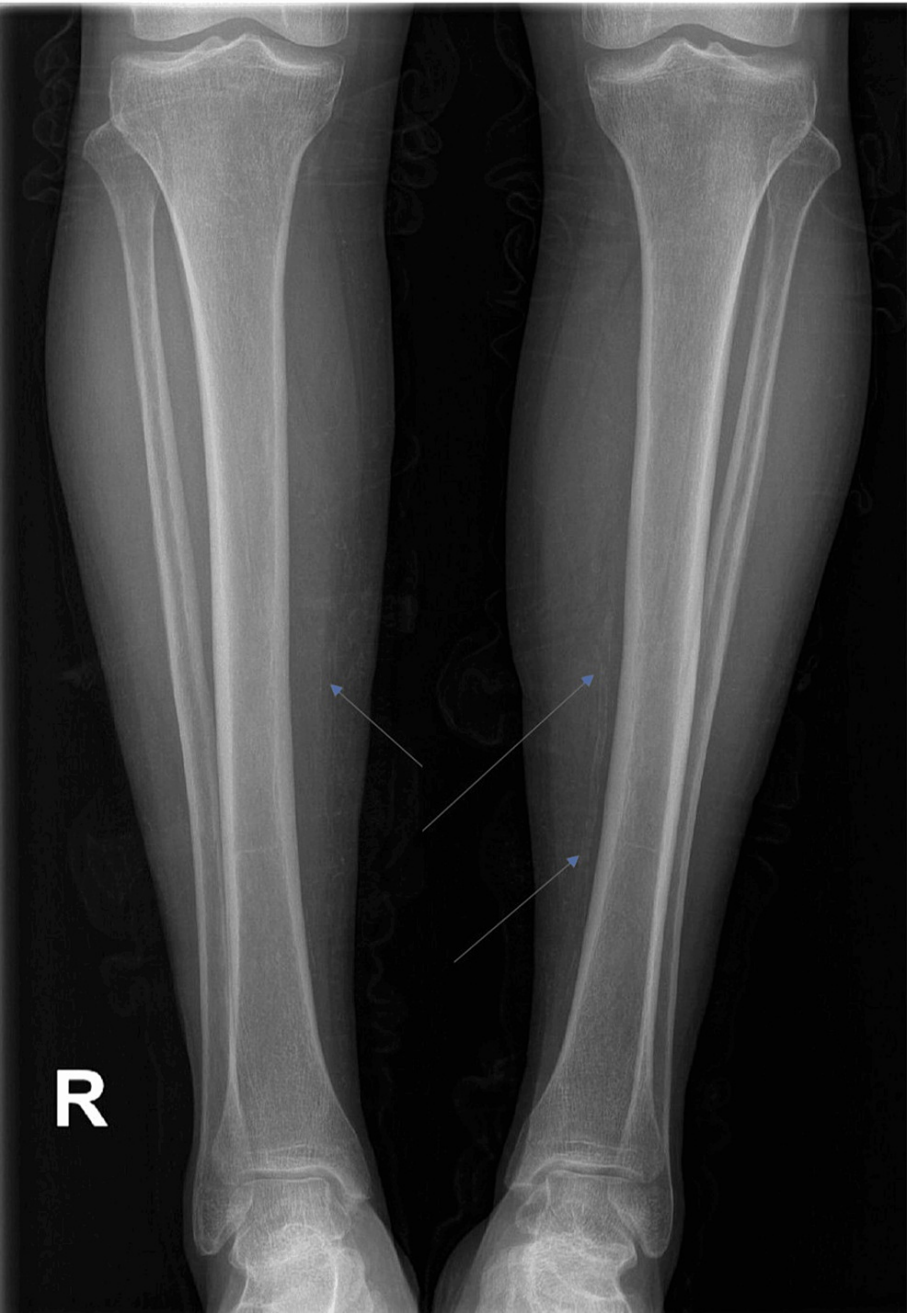
Calcifications along vascular pathways

The radiological examination of the lower limbs revealed calcifications along vascular pathways, supporting the clinical suspicion of calciphylaxis. Specifically, vascular wall calcifications were identified at the level of the tibial arteries and were predominantly evident on the left side (Figure 2).

Thus, the suspicion of calciphylaxis was confirmed based on the highly suggestive clinical presentation and radiological findings. Consequently, a skin biopsy with histopathological examination to confirm the diagnosis was not considered necessary.

During the patient's hospitalization, an anti-inflammatory regimen comprising systemic corticosteroids (prednisone 20 mg/day) was initiated, with doses gradually tapered over time. Concurrently, systemic antibiotic therapy with cephalexin (1 g daily) along with analgesics for pain management (tramadol 300 mg daily) and medication for the treatment of associated pathologies such as diabetes (gliquidone 30 mg twice daily) and hypertension (amlodipine 10 mg /day, clonidine 0.15 mg/day). In addition, silver sulfadiazine cream and anesthetic cream containing lidocaine and prilocaine were topically applied. The patient continued hemodialysis sessions as scheduled with a stable evolution of calciphylaxis in the absence of any specific treatment. Unfortunately, sodium thiosulfate, which is considered one of the therapeutical agents that can stop the evolution of this disease, is not available in our country. We recommended surgical debridement, but the patient refused any invasive intervention. Despite this, her condition has remained stable for two months of follow-up.

## Discussion

Calciphylaxis is a rare multifaceted ischemic vasculopathy with diverse etiological risk factors that is typically observed in patients with renal disease. It is characterized by vascular calcification and cutaneous necrosis, which result in a high mortality rate, with more than 50% of patients succumbing within one year of diagnosis. Calciphylaxis predominantly affects the lower extremities. The diagnostic histopathological feature is the intravascular deposition of calcium in the media of dermal and subcutaneous arterioles. The treatment options for calciphylaxis include wound care, surgical debridement, sodium thiosulfate, bisphosphonates, and hyperbaric oxygen [5,6].

The precise pathogenesis of calciphylaxis remains unclear. However, low levels of vascular calcification inhibitors may play a role in the development of calciphylaxis. Specifically, fetuin-A, osteoprotegerin, and Matrix G1a protein have been identified as key inhibitors that are downregulated in dialysis patients. Abnormal differentiation of vascular smooth muscle cells into an osteoblast-like phenotype has also been observed. Fetuin-A is a glycoprotein that binds circulating calcium and phosphorus, while Matrix G1a protein is produced by vascular smooth muscle cells and is vitamin K-dependent. Both proteins play a role in preventing the calcification of blood vessels and soft tissues. Warfarin administration interferes with the vitamin K-dependent activation of Matrix G1a protein and can lead to the development of calciphylaxis [5-7]. There is also a medical entity called warfarin-induced skin necrosis, presenting a similar clinical picture with ulcerative-necrotic plaques. Warfarin-induced skin necrosis is extremely painful, non-healing, and is predominantly located in adipose-rich areas. Notably, warfarin-induced necrosis occurs within a few days after initiation of warfarin and typically resolves following discontinuation of the drug. In contrast, warfarin-induced calciphylaxis appears later and takes longer to resolve after cessation [8].

Regarding our patient, a differential diagnosis of cutaneous vasculitis with arterial ulcerations was considered. Cutaneous vasculitis can progress from painful purpuric lesions to necrotic, black-colored plaques with an active purpuric edge, a feature absent in the present case. The mechanism leading to necrosis involves thrombosis of the dermal vessels. Subsequently, ulcerations develop, varying in size and depth depending on the blood vessels involved, representing the final stage of disease progression. Additionally, we considered arterial ulcers in the context of diabetes, atherosclerosis, or hypertension, where circumferential calcification of tibial vessels is present. During Doppler ultrasound examinations, changes in the arterial waveform and decreased pulse volume recordings can be observed. As arterial impairment progresses, the Doppler signal becomes monophasic over time. However, no discernible changes were noted during the arterial Doppler examination in this particular case.

In calciphylaxis, the initial clinical manifestations include indurated subcutaneous nodules or plaques associated with livedo reticularis, mimicking dermo-hypodermitis. These lesions evolve within a few days, from superficial ulcers to deep ulcers with a black eschar appearance, extremely painful, and with a centrifugal distribution. Clinical suspicion is raised by the painful clinical manifestations, accompanied by palpation of firm subcutaneous nodules and suggestive ulcerative and necrotic skin lesions seen in dialysis patients or those with other risk factors for calciphylaxis. A complete patient history and a thorough physical examination are necessary to detect additional lesions. In anticoagulated patients, differentiation between calciphylaxis and warfarin-induced necrosis is essential [5-8].

Effective management and treatment are crucial after calciphylaxis diagnosis to alleviate the patient's suffering, prevent further complications, and reduce mortality rates. A comprehensive approach to treatment is necessary, which requires collaboration among nephrologists, dermatologists, plastic surgeons, wound care specialists, and nutritionists. Pain management strategies typically involve the use of pain relievers, proper wound care, ensuring adequate nutrition, eliminating iatrogenic factors such as warfarin, and proactively treating predisposing conditions. Additional therapeutic options that may be considered include sodium thiosulfate, bisphosphonates, and hyperbaric oxygen. The focus of management primarily revolves around local wound care and preventing both local and systemic infections. At the same time, efforts are directed towards optimizing medical therapy for any associated comorbidities [9,10].

## Conclusions

Healthcare practitioners should remain vigilant for the potential occurrence of calciphylaxis in patients with end-stage renal disease who present with painful cutaneous lesions. Calciphylaxis, a condition characterized by significant morbidity and mortality, commonly presents with calcification in the medial arteriolar layers, as revealed by skin biopsy and histopathological examination. However, alternative diagnostic approaches may be necessary, given the risks associated with skin biopsies, including lesion propagation, ulceration, superimposed infection, necrosis induction, and bleeding. In cases where performing a skin biopsy is not feasible, the identification of calcifications along vascular pathways through radiological examinations and a thorough clinical evaluation is sufficient for diagnosis. 

It is worth noting that certain medications have been identified as potential risk factors for calciphylaxis, emphasizing the significance of conducting a complete review of patients' medication history when calciphylaxis is suspected. In addition, the management of calciphylaxis requires a multidisciplinary approach comprising nephrologists, dermatologists, wound care specialists, and nutritionists. 
